# Estimation of overlapped Eye Fixation Related Potentials: The General Linear Model, a more flexible framework than the ADJAR algorithm

**DOI:** 10.16910/jemr.10.1.7

**Published:** 2017-10-07

**Authors:** Emmanuelle Kristensen, Bertrand Rivet, Anne Guérin-Dugué

**Affiliations:** 1 Univ. Grenoble Alpes, GIPSA-Lab, F-38000 Grenoble France CNRS, GIPSA-Lab, F-38000 Grenoble France; Univ. Grenoble Alpes, GIPSA-Lab, 11 rue des Mathématiques Grenoble Campus, BP 46, 38000 Grenoble France; GIPSA-Lab

**Keywords:** Eye Fixation Related Potential, General Linear Model, ADJAR algorithm, eye movement, eye tracking

## Abstract

The Eye Fixation Related Potential (EFRP) estimation is the average of EEG signals across epochs at ocular fixation onset. Its main limitation is the overlapping issue. Inter Fixation Intervals (IFI) - typically around 300 ms in the case of unrestricted eye movement- depend on participants’ oculomotor patterns, and can be shorter than the latency of the components of the evoked potential. If the duration of an epoch is longer than the IFI value, more than one fixation can occur, and some overlapping between adjacent neural responses ensues. The classical average does not take into account either the presence of several fixations during an epoch or overlapping. The Adjacent Response algorithm (ADJAR), which is popular for event-related potential estimation, was compared to the General Linear Model (GLM) on a real dataset from a conjoint EEG and eye-tracking experiment to address the overlapping issue. The results showed that the ADJAR algorithm was based on assumptions that were too restrictive for EFRP estimation. The General Linear Model appeared to be more robust and efficient. Different configurations of this model were compared to estimate the potential elicited at image onset, as well as EFRP at the beginning of exploration. These configurations took into account the overlap between the event-related potential at stimulus presentation and the following EFRP, and the distinction between the potential elicited by the first fixation onset and subsequent ones. The choice of the General Linear Model configuration was a tradeoff between assumptions about expected behavior and the quality of the EFRP estimation: the number of different potentials estimated by a given model must be controlled to avoid erroneous estimations with large variances.

## Introduction

Seminal works (19); (3) combined electrooculography (EOG) and electroen- cephalography (EEG) to gain understanding of the consequences of eye movements on EEG activity. From their work, the Eye-Fixation Related Potential (EFRP) technique was developed to provide greater insight into mechanisms related to eye movements, and the time course of the continuous cognitive processing involved in experi- mental tasks. This technique requires joint electroenceph- alography (EEG) and eye-tracking acquisition. Its fundamental difference from the popular Event-Related Potential (ERP) technique is that the neural response extracted is synchronized with ocular fixation rather than with the onset of a stimulus. In the context of a visual exploration experiment, for example, the ERP is the neural response that is time-locked with image onset, whereas the EFRP is the neural response elicited at fixation onset. When the EFRP technique is employed, the cognitive processes involved and their timelines are explored by encoding visual information through tasks of greater ecological validity (everyday tasks). During a classical ERP experiment, participants are required to gaze at a given location on the screen to avoid eye movement artifacts, whereas in an EFRP experiment they can explore the visual scene freely. During a reading experiment using the ERP approach, for instance, the text is displayed word by word on the screen in the same location to avoid ocular artefacts, whereas in the EFRP approach, the whole text is displayed at the same time and participants can move their eyes freely (15), (17).

EFRP estimation is based on the average across epochs at fixation onset. Because the Signal to Noise Ratio is low, the visual task is repeated several times to provide a sufficient number of epochs. However, the average obtained leads to an unbiased EFRP estimation if, and only if, a single potential is evoked inside an epoch. This condition cannot often be fulfilled when estimating EFRP. Inter-Stimuli Intervals can be controlled with ERP technique. By contrast, Inter-Fixation Intervals (IFI), which are the sum of the duration of a current fixation and of the subsequent saccade, depend on the oculomotor pattern of each participant. IFI duration (typically around 300 ms) can be shorter than the latency of the components of the evoked potentials. Overlap between several evoked potentials inside a single epoch is, therefore, a major limitation of the EFRP technique (15); (1); (29). Different strategies have been adopted to address this issue:

• In (24), participants were trained to fix the target for one second. In this way, only one fixation occurred per epoch. However, this strategy reduces the ecological validity of the protocol and can only be used in specific experiments.
• In (5) in a visual search task, and (32) in a guided search task, fixations shorter than 500 ms were excluded from the analysis on the P300 component (an epoch lasted 500 ms to be free of eye movements). In (23) a similar selection was implemented for the same reason, with a threshold of 550 ms, in a visual search task. Only a part of all epochs recorded is used to estimate the EFRP. Data loss is a drawback in this procedure, which is an ad-hoc procedure designed to avoid eye movement within the latency window of the component of interest (component P300 in the three studies above).
• The matching of eye movement characteristics is the most common strategy (11); (12); (14); (16); (23); (29). This technique compares ocular data that are similar in size, direction, duration, etc. It allows the distortions due to overlap between different experimental conditions to be counterbalanced, but not corrected.
• In addition to the matching technique, Dias and colleagues used a subtraction technique to correct distortions due to saccade response overlap (14). Dandekar and colleagues (9) also developed a well-known linear regression method, the General Linear Model (GLM) (26); (8) to extract the potentials elicited by ocular saccades of different sizes and orientations which were affected by the overlapping issue.
• In (27), a regularized GLM was studied and compared to the classical estimation by averaging, and was evaluated to estimate EFRP during the free exploration of visual scenes, irrespective of fixation rank.

EFRP estimation is the central question in this study. It requires the correction of distortions due to response overlaps elicited by adjacent fixations. We addressed this question from two perspectives.

In the first of these, we compared three classical algorithms in a common framework where identical evoked potentials overlapped. In order to do so, we compared these three approaches using real data from joint EEG and eye-tracking recordings during a free visual exploration experiment: (1) the classical estimation by averaging time- locked EEG signals, (2) the popular Adjacent Response (ADJAR) algorithm (40), developed for the ERP technique and (3) the GLM configured to deconvolve evoked potentials with temporal overlaps. These algorithms were compared to estimate the potentials elicited by fixations in the middle of visual exploration, with the assumption that potential was the same irrespective of fixation rank. Moreover, in accordance with this aim, these algorithms were compared on their ability to deconvolve identical overlapped potentials, and not on their ability to estimate different kinds of potential per epoch.

In the second perspective, based on these results, two case studies focused on the GLM in order to choose the best match between GLM configuration and targeted objectives. Unlike in the first perspective, the GLM was used to estimate not only a unique potential, but two or three different potentials. The two case studies deal with the estimation (i) of the EFRP at the first fixation onset and (ii) of the ERP at image onset. In methodological studies (15), (29), specifying the first EFRP was recommended for at least two reasons: (1) differences in ocular features, (2) influences of neural activity at stimulus onset. In line with these recommendations, in the first case-study, a three-class GLM was implemented to estimate the first EFRP compared to the potential elicited by the second and following fixations. In this case-study, the potential elicited by image presentation was also included in the model since this potential overlaps the EFRP at the very beginning of exploration. The potential evoked at image presentation was estimated in the second case study. We were interested in the impact of the differentiation of the first fixation on the estimation of the ERP at image onset. To study this, the three-class GLM was contrasted with the two-class GLM, in which only the ERP at image onset and the EFRP irrespective of fixation rank were estimated. The aim of these two case-studies was to show that an efficient estimation by GLM is produced by a tradeoff between assumptions of expected behavior and the model’s parsimony: the number of different potentials (i.e. number of classes for the GLM configuration) estimated by a GLM must be carefully set to avoid a large estimation variance. It is the expression of the classical tradeoff between bias and variance.

## Materials and Methods

In this Section, the methods used to estimate the EFRP are detailed, then a description of our experimental data follows.

### Eye Fixation Related Potential Estimation

We present three methods of estimating EFRP: the classical average of epochs, known as the Average method, the ADJAR algorithm in the context of EFRP and the GLM. To avoid confusion, we define here the main terms of the description of algorithms. Depending on the objective of the EFRP study, a temporal interval of interest, called the *window of interest*, has to be decided. This interval can, for example, be between -200 ms and 600 ms to include activities before fixation onset (saccadic potentials), and also to include activity for early and late components. In order to do so, EFRP is estimated during the *estimation window*, which may be either the same interval or a larger one which includes the window of interest, depending on the estimation method, as described below. EFRP estimation is based on a set of epochs time-locked on the event of interest (stimulus presentation, fixation onsets, or saccade onsets). The time interval of the epoch *(epoch window)* therefore has to be defined in relation to the estimation window. The choice of the other two intervals (estimation window and epoch window) will be explained for each of the three methods in accordance with the window of interest.

#### Average estimation and overlapping issue

Let us consider the given *i^th^* fixation during a trial. The observed neural response *x_i_*(t) time-locked on this *i^th^* fixation onset can be written as:


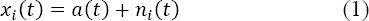


where *a(t)* is the potential evoked at this fixation onset, and *n_i_(t)* a noise corresponding to ongoing brain activity. The estimation of EFRP *a(t)* by averaging is based on the implicit assumption that the neural potential *a(t)* is the same for each eye fixation. For each repetition, the signal is time-locked at fixation onset, segmented into epochsand then averaged. The temporal epoch window must be long enough to include the latency and the whole temporal evolution of the potential of interest. The neural response *x_t_ (t)* is observed throughout the epoch. The EFRP *a(t)* is estimated by averaging all epochs. Thus, for the estimation by averaging, there is no distinction between the temporal epoch window, the estimation window and the window of interest. The number of epochs must be large enough to significantly cancel out ongoing brain activity and to increase the Signal to Noise Ratio. Let *E* denote the number of epochs^1^. In this way, *a(t)* is estimated by averaging across these epochs:


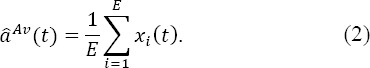


With equation (1), only a single evoked potential per epoch is taken into account. Consequently, this model provides an unbiased estimator if IFI duration is longer than the latency window of the components of interest in the evoked potential. If this condition is not fulfilled, more than one fixation occurs inside the same epoch, there is some over-lapping between adjacent responses and the estimation is biased. To take these overlaps into account in a linear way, equation (1) is rewritten as:


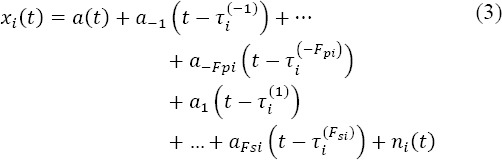


where *F_pi_* (respectively *F_sl_)* is the number of previous (resp. subsequent) fixations which occur during the *i^th^* epoch. *a_f_(t)* is the potential elicited at the *f^th^* fixation’s onset *(f > 0* means subsequent and *f <0* means previous). 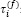 is the timestamp of the *f^th^* fixation: 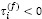, and *f = -1,…,-Fpi* for previous fixations and 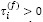 and *f = l,…,F_sl_* for subsequent fixations inside the *i^th^* epoch. By averaging across the epoch, equation (3) highlights a summation of convolution products (*) between the evoked potentials and the normalized distribution of the timestamps 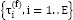:


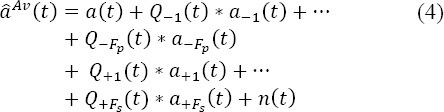


where 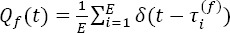 is the normalized^2^ distribution of the timestamps at the onset of the *f^th^* rank fixation and *n(t)* is the average of ongoing brain activity 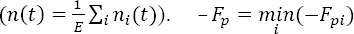 (respectively 
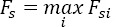
) is the minimum (respectively maximum) rank of the previous (resp. subsequent) fixations across all epochs. This last equation can be recast in the following compact form:


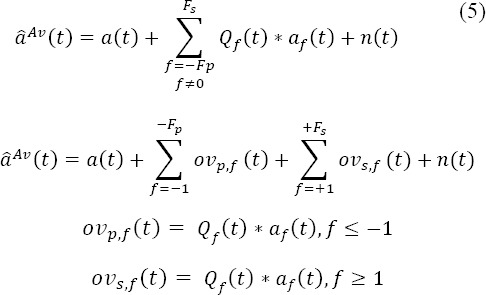


where *0v_Pjf_(t)* (resp. *ov_S,f_(t))* is called the previous (resp.subsequent) response overlaps. This equation highlights the overlapping issue: i.e. the term 
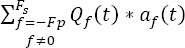
 does not necessarily decrease to *0* even if *E* increases to infinity.

The next two subsections present the ADJAR and GLM methods designed to deal with the overlapping issue.

#### Estimation by the ADJAR algorithm

In the context of ERP estimation time-locked on external events, Woldorff proposed the ADJAR algorithm (40) to correct distortion of the ERP due to the overlap from temporally adjacent responses. In this article, the algorithm is described in the context of EFRP estimation.

The underlying assumptions are:

1. Each evoked potential is identical, irrespective of fixation rank (i.e. *a_-1_(t) = a_+1_(t) = a(t)*, with *a(t)*, the underlying response);
2. During each epoch, only overlaps from immediately adjacent fixations are considered (i.e. *|f|* = 1). In other words, during each epoch, overlaps from earlier and later fixations are not considered (i.e. when|*f*| > 1);
3. At the second-order, the amplitude of higher order adjacent responses is already considered to be negligible (Q_+1_(t) * *Q*_+1_(t) * a(t), *Q*_-1_(t) * *Q*_-1_(t) * a(t)). See below for details;
4. Noise due to ongoing activity is not considered.

Based on these assumptions, equation (4) becomes:


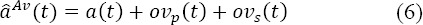


where *ov_p_(t) = ov_p-1_(t)* and *ov_s_(t) = ov_s+1_(t)* are called the first-order, previous, and subsequent response over-laps, respectively. It is important to note that Ģ*_-1_(t)* and Q*_+1_(t)* are normalized (by the number of epochs): for each of them the integral of their contribution is equal to one. The distribution *Q*_-1_(t) (resp. *Q*_+1_(t)) is computed from the timestamps of the previous (resp. subsequent) fixation extracted from each epoch. The epoch must be sufficiently large to include both the previous and the subsequent fixation onsets. The estimation window for the ADJAR algorithm must be slightly larger than the window of interest, to take into account the border effects of the iterative procedure using convolutions. From a practical point of view, a tapering window is applied at each iteration step to manage these border effects. Schematic illustrations of these windows, describing the ADJAR algorithm implementation in this study, are shown in the appendix.

From the distributions *Q*_-1_(t) and *Q*_+1_(t), the first-order response overlaps *ov_p_(t)* and *ov_s_(t)* are estimated itera- tively. At each iteration, these estimates are then subtracted from the average *â^Av^(t)*, to progressively improve the estimation of *a(t)*. After the *k^th^* iteration, the estimations of response overlaps are updated as: 


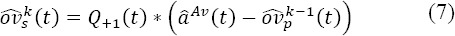



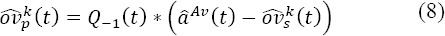


with 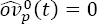, as the initialization for the first iteration. The estimation of *a(t)* after the *k^th^* iteration is given by:


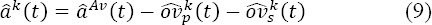


The iterative procedure stops when the estimate *â^k^(t)* no longer changes between two consecutive iterations. Let 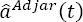 denote the estimate given at the last iteration. The development of the earlier iterations is shown in the ap pendix. As a result, the final estimation is equal to:


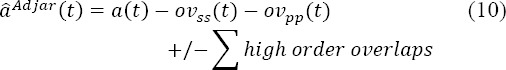


where ov_pp_(t) = *Q*_-1_(t) * *Q*_-1_(t) * a(t) = *Q*_-1_(t)^(^*^2)^ * a(t) and ov_ss_(t) = *Q*_+1_(t)^(^*^2)^ * a(t) denote second-order response overlaps. Other terms appear in the iterative development of *â^Adjar^(t)*, for example: Q_-1_(t)^(*k)^ * *Q*_+1_(t)^(^*^k)^ * a(t). They are called higher order response overlaps, and contribute positively or negatively to the final estimation. The ADJAR algorithm estimates and iteratively removes first-order response overlaps. Thus only second and higher order response overlaps remain. However, upon successive convolutions, higher order response overlaps are naturally progressively shifted outside the estimation window as the iteration number increases. Consequently, convergence is confirmed if convolution shifts the distributions of the second-order terms (Q_-1_(t)^(^*^2)^ and *Q*_+1_(*t*)^(^*^2)^) far enough outside the estimation window to render them negligible for all following iterations. In other words, convergence towards the underlying potential *a(t)* is only possible if distortions in the measurements are due solely to first-order response overlaps. This assumes that the second-order response overlaps are negligible. These conditions are in line with assumptions 2 and 3 presented above. However these conditions may or may not be fulfilled, as they depend on the temporal ranges of the distributions *Q_-1_(t)* and *Q*_+1_(*t*) in relation to the epoch duration. This condition will be discussed in the context of EFRP in the section entitled “Estimation of EFRP during exploration”. Assumptions 2 and 3 are, moreover, directly linked to convergence towards an unbiased estimation of the evoked potential. In the appendix, we show how these assumptions constitute strong limitations of the ADJAR algorithm. A modified ADJAR algorithm was designed to take into account previous and subsequent events within an epoch, but the iterative procedure did not converge.

The ADJAR algorithm is a trade-off between the accuracy of the model and the quality of the estimation. On the one hand, using only first-order response overlaps provides a convergent algorithm leading to a biased estimator of the response overlaps. On the other hand, taking into account all contributions to ensure better modeling of the response overlaps leads to a divergent algorithm.

#### Estimation by the General Linear Model

The GLM (26) (8) was proposed to manage the overlapping issue, taking into account all fixations that occur during an epoch.

Equation (3) can be rewritten in a matrix form:


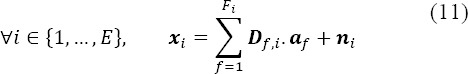


where *x_i_* represents EEG samples observed during the *i^th^* epoch, 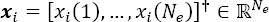 with *N_e_* the number of samples and 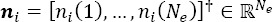 with .^†^ the transpose operator. 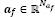 is the vector of the response time-locked on the onset of the *f^th^* fixation and *N_a_f__* is the length of the response *a_f_(t)*. Each potential *a_f_(t)* is esti- mated within an estimation window equal to the chosen window of interest. *F_t_* is the number of fixations inside the epoch. Finally, 
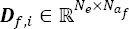
 is the Toeplitz matrix for the *f^th^* fixation and the *i^th^* epoch, defined by its first column with entries that are all equal to zero except which 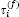 is equal to one, with 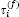 the onset of *f^th^* fixation in the *i^th^* epoch. Consequently, all epochs can be concatenated to obtain:


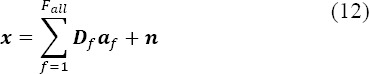


with *x_i_* equal to 
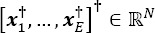
 and ***D***_*f*_ equal to 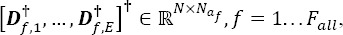, where is the total number of samples and *Ν = N_e_ χ E* and *F_all_* the total number of fixations.

Equation (12) can be recast as:


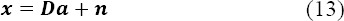


with *D* equal to *[D_1t_ …,D_Fall_]* and a is the concatenation of the evoked potentials such as 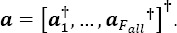. The solution given by the least square minimization is:


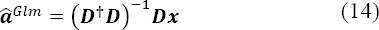


where 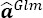 is the concatenation of all estimates, such as 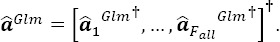


The GLM configuration is presented with *F_all_* fixation classes according to their rank inside the epoch. However, the fixations can be grouped in different ways depending on the assumptions made about the differentiation of the potentials evoked by consecutive fixations during the experimental task.

It is well known that the quality of estimation by the GLM is linked to the condition number of the ***D***^†^***D*** matrix that has to be inverted to obtain the final estimation (equation (14)). The higher the condition number is, the more singular the ***D***^†^***D*** matrix is, and consequently the less accurate the estimation. Conversely, the condition number is small if ***D***^†^***D*** is a diagonal dominant matrix. Using GLM, Bardy and colleagues (2) showed that the condition number of the ***D***^†^***D*** matrix was linked to the amount of jitter on the Inter Stimuli Intervals in their experiments using sequences of auditory stimuli. In the context of our study, this result applies to the amount of jitter between the timestamps 
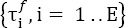
 of events between each class.

Two cases will be considered in the following sections: the estimation of EFRP in the middle of visual exploration, and that of evoked potentials at the beginning.

### Experimental data

Real data was used from an experiment on the free exploration of natural scenes in order to evaluate EFRP estimation using the three methods described in the previous subsection. Three conditions were included in the experiment: free exploration, image categorization, and visual search (12). However, for the purpose of this study, only the condition involving free exploration was used.

#### Participants

Thirty-nine healthy adults participated in the experiment (22 women and 17 men; age range: 20-36; mean = 24.69; std = 3.49). The data from five other participants were removed because of technical problems during the recording process. All participants had normal or cor- rected-to-normal vision. The study was reviewed and approved by the local French ethics committee of the “Pôle Grenoble Cognition”. All participants gave their written and informed consent prior to the experiment.

#### Apparatus

Visual scenes were displayed on a 20-inch ViewSonic CRT monitor located 57 cm from the participants, with a resolution of 768× 1024 pixels and a refresh rate of 75 Hz. Scenes subtended 30×40 degrees of visual angle.

Eye movements were recorded with a video-based infrared eye-tracking system (EyeLink® 1000, SR Research Ltd., Ontario, Canada) and sampled at 1000 Hz, for both eyes. The head was stabilized using a chin rest. A 9-point calibration routine was carried out at the beginning of each session and was repeated every 20 trials or when the drift correction, performed every 10 trials, reported a mean error above 0.5°.

The EEG activity was recorded using 32 Ag/ AgCl unipolar active electrodes positioned according to the extended 10-20 system (22). The right earlobe and FCz electrode were used respectively as reference and ground. Data were amplified using a g.GAMMAsys gtec system (g.tec, Inc.) and sampled at 1200 Hz using the g.USBAmp (g.tec, Inc.). An analog band-pass filter (0.01-100 Hz) and a 50 Hz notch filter were applied online.

#### Stimuli

The stimuli consisted of 240 color pictures (of various indoor and outdoor scenes). The scenes did not contain any images of people.

#### Experimental procedure

Participants performed four 20-minute sessions, but only the results for one session, (free exploration condition) are discussed here. Sixty scenes were randomly displayed within each session.

The experiment was designed using the SoftEye software (21) to control (i) the timescale for displays, (ii) the eye-tracker and (iii) the sending of synchronization triggers to both devices, i.e. the EEG and eye-tracker. In the free exploration condition, trials were composed of three successive displays. Each trial started with a white central fixation cross, which was displayed for 800 to 1200 ms. Once participants had stabilized^3^ their gaze for 100 ms on a square of 50 pixels around the central fixation cross, a scene was displayed for 4 s. Each trial ended with a grey screen for 1 s.

### Data preprocessing

Saccades, and consequently fixations, were automatically detected by the Eyelink software using three thresholds: velocity (30 °/s), acceleration (8000 °/s^2^) and sac- cadic motion (0.15 °). We analyzed the data for the dominant eye of each participant, and only fixations of between 50 and 1000 ms were retained for analysis.

Eye movement and EEG signals were synchronized offline, on the basis of triggers sent simultaneously to both the EEG system and the eye-tracker. EEG data were then re-sampled at 1000 Hz (eye-tracker sampling rate). Visual inspection revealed that channels T7, T8, TP9 and TP10 were too noisy for the majority of participants. We therefore decided to cancel these four channels for all participants. Using EEGlab software (11), EEG data were segmented into trials ranging from 500 ms before scene onset to 4000 ms afterwards. The segments obtained were visually inspected offline, and those containing muscular activity or non-physiological artifacts were rejected. Ocular artifacts were then corrected using a principal component analysis (number of channels minus one component) followed by ICA (infomax ICA) (4). A visual inspection was performed once again. If ocular artifacts had not been corrected, epochs were removed from the analysis. One participant was excluded because of a high number of deleted trials (37). Four other participants were removed because of high variance across trials. The individual inter-trial variance (averaged on times and channels) was computed for each subject. Four subjects had the highest individual average variance with a relative gap of more than 60% with the average variance. This was confirmed by the empirical variance of these individual variances across subjects^4^. In the end, trials from thirty four participants were retained for analysis. On average, 47.7 ± 6.3 trials were kept for analyses. The maximum number of trials per participant was 57, and the minimum number was 30.

Fixations were tagged off-line according to their rank, from the first to the last fixation. The beginning of the first fixation started after the scene onset, and the last fixation was such that this fixation ended before the scene offset.

## Results

This section is divided into three parts. The first part presents the behavioral results which were used as a basis to cofigure estimation methods for the evoked potentials. The second part deals with the comparison between the linear regression method (GLM) and the ADJAR algorithm to estimate EFRP in the middle of the free visual exploration task. Finally, and using the same datasets, the results in which the GLM is configured to estimate potentials evoked at the beginning of exploration, i.e. the potential elicited at image onset, as well as the EFRP at the beginning of exploration are presented in the third part. Based on the results presented in part two, the ADJAR algorithm was not implemented for comparison in part three.

### Behavioral results

Firstly, we present the behavioral results on ocular activity across the whole trial, and then at the start of visual exploration, i.e. for earlier fixations.

#### Eye movement during the free exploration task.

Table 1 summarizes the averages of the main ocular features evaluated throughout the entire duration of the trials (number of fixations, first saccade latency, fixation duration, saccade amplitude, saccade duration, inter-fixation interval duration 4 s).

**Table 1:** Statistical summary: number of fixations, first saccade latency, fixation, saccade and Inter-Fixation Interval durations and saccade amplitude, based on individual means (std), during free exploration

Number of fixations	First saccade latency [ms]	Fixation duration ^[ms]^	Incoming saccade amplitude [°deg]	Incoming saccade duration [ms]	IFI duration [ms]
12.60 (1.27)	266.85 (44.57)	243.40 (26.29)	7.18 (0.97)	45.06 (6.48)	288.69 (26.97)

#### Eye movement on the first fixations

Table 2 summarizes the averages of the main ocular features, but only for the first five fixations. These early fixations were analyzed specifically because, in the section entitled “Estimation by GLM of evoked potentials at the beginning of exploration”, we were interested in the potential evoked at image onset, i.e. at the very beginning of the task.

**Table 2:** Statistical summary for the first five fixations: latency, fixation duration, amplitude and duration of the incoming saccade, based on individual means (std), at the start of free exploration

Rank / Feature	Fixation latency [ms]	Fixation duration [ms]	Incoming saccade amplitude [°deg]	Incoming saccade duration [ms]
Fixation 1	307.65 (46.14)	209.57 (38.93)	5.03 (0.79)	40.80 (9.52)
Fixation 2	561.50 (80.85)	241.98 (36.40)	6.46 (1.13)	44.30 (13.69)
Fixation 3	847.58 (108.18)	243.65 (26.03)	6.28 (1.26)	43.06 (12.11)
Fixation 4	1135.42 (126.89)	243.01 (20.56)	6.94 (1.39)	43.15 (7.58)
Fixation 5	1421.69 (145.30)	241.76 (26.21)	7.40 (1.38)	44.25 (7.78)

A repeated measures analysis of variance was used, with fixation rank (five ranks) as the within-participant factor, and multiple comparisons were assessed using the Bonferroni correction. The statistical results on fixation duration revealed that this was significantly lower for the first rank than for subsequent ranks (F(4, 165) = 8.04, p < 0.001). The same result was observed for the incoming saccade amplitude determined from ranks one to five (F(4, 165) = 18.35, p < 0.001); incoming saccade amplitude for the first fixation was smaller than for the following ones. But the differences in saccade duration across the rank were not significant (p = 0.64). It has been established that a saccade’s duration is linearly related to its amplitude (39). We therefore expected a significant difference for the first saccade duration. The result obtained could be explained by the fluctuations in individual correslations between duration and amplitude observed. This provided a relative standard deviation (ratio of the standard deviation to the mean) that was larger for the duration than for the amplitude of the first saccade.

To sum up, we found that the first fixation differed from the following ones in terms of fixation duration and incoming saccade amplitude. Free exploration of a scene began with a fixation cross at the image’s center. At the very beginning of the exploration, the first fixation remained close to the image’s center^5^, contributing to the so- called central fixation bias usually observed in scene viewing (36).

#### Luminance and contrast at the first fixations

The early potential (lambda component) is modulated by the physical properties of the regions gazed at (18), (20), (31). Table 3 summarizes the average image features evaluated for the regions gazed at during the first fixations. Three luminance features were evaluated based on the region gazed at during each fixation. The features were evaluated for regions restricted to the foveal region: the average luminance, the contrast computed by the standard deviation of the local luminance of the fixated region and the absolute value of the difference of the mean luminance between two regions on consecutive fixations (for a given fixation the absolute value of the luminance difference lies across the incoming saccade).

**Table 3:** Statistical summary for the first five fixations, of the image features of the fixated region: mean local luminance, local contrast computed by the standard deviation of the local luminance and absolute value of the difference of the mean luminance of fixated regions before and after the incoming saccade, based on individual means (std), at the start of free exploration

Rank / Feature	Luminance	Contrast	Luminance difference on saccade
Fixation 1	115.09 (7.54)	44.34 (3.02)	42.27 (4.13)
Fixation 2	112.78 (8.44)	41.99 (3.07)	42.90 (5.48)
Fixation 3	114.58 (7.72)	41.42 (2.38)	41.25(7.07)
Fixation 4	115.63 (6.74)	42.29 (2.43)	41.65 (6.97)
Fixation 5	113.98 (7.13)	41.99 (3.10)	41.79 (5.84)

A repeated measures analysis of variance was used with fixation rank (5 ranks), as within-participant factors, and multiple comparisons were assessed using the Bonfer- roni correction. Statistical results of the local luminance standard deviation revealed that the differences across the fixation ranks were significant (F(4, 165) = 5.42, p < 0.001): the standard deviation of the luminance of the first region gazed at during the first fixation was significantly higher than that of subsequent fixations. On average, for all trials and subjects, the local statistics on the luminance (mean and contrast, evaluated here by standard deviation) were similar for all fixation ranks except the first one. The local contrast at the first fixation position was higher than at later ones. This first fixation was located near the screen center and the higher contrast might be explained by bias due to the center driven composition of usual image databases.

### Estimation of EFRP during exploration: comparison between Average, ADJAR and GLM estimations

In this section, we present the results of the comparison of the three methods of EFRP estimation during a free vis-ual exploration task. Firstly, the ADJAR algorithm is ana-lyzed alone to provide details of its estimation procedure. We go on to compare three estimates of the EFRP: (i) the ADJAR algorithm, (ii) the GLM and (iii) the classical average on time-locked signals, as a baseline method, even though it is known that this last estimation is biased by overlap.

#### Explanation of the configuration of the three algorithms

The three methods were compared only in relation to the overlapping issue, and not in relation to their ability to tackle different kinds of potentials per epoch. To set a fair benchmark for the three methods (Average, ADJAR and GLM), the epochs were selected in the middle of visual exploration. This ensured that the hypothesis of a single EFRP elicited irrespective of fixation rank was acceptable.

The following methodology was designed:

- For the estimation method by average and in line with the basic requirement for the ADJAR algorithm, it was assumed that each fixation elicited the same potential regardless of its rank during exploration. During the free exploration of these scenes, there were no specific spatial loci (e.g., for instance people, faces, incongruent objects, etc.) which could elicit specific potentials. Only fixations in the middle of visual exploration were selected (see below).
- The observed *x_t_* signal for the *i^th^* trial was time-locked at the onset of the *n^th^* fixation. For each participant and each scene presentation, a rank *n* was randomly selected from 3 to 9 three^6^ times, on the basis of a uniform draw. The delay between the onset of the visual stimulus and the onset of the first fixation should be large enough to allow the temporal overlap between the potentials evoked by these two events to be ignored. Based on methodological studies (15); (29), a minimum delay of 700 ms was chosen and the choice of the third fixation led to an average (std) latency of 847.58 (108.18) ms, justifying the lower bound value of 3. Moreover, a ninth fixation with an outgoing saccade on the visual scene before the end of the trial occurred in all trials, justifying the upper bound value of 9. This choice resulted in an average (std) number of epochs per participant of 165.26 (7.11).
- The window of interest for the EFRP ranged from - 250 ms to 600 ms in relation to fixation onset. This window is of a typical configuration, designed to evaluate the waveform both before the saccade and after the saccade for the early and late components of the EFRP. For the estimations by average and by GLM, the EFRP (*a^Av^(t)* and *a^Glm^(t)* respectively) were then estimated on an estimation window equal to the window of interest. However, the estimation window for the ADJAR algorithm was slightly larger to include the border effect of the convolution product with a tapering window. For the EFRP *a^Ad^’^ar^*, the estimation window was therefore defined as being from -450 ms to 800 ms in relation to fixation onset. The parametrization of the algorithm is shown in the appendix describing the ADJAR implementation for this study.
- Accordingly, for the estimation by average, the epoch window was set to the chosen estimation window (i.e. from -250 ms to 600 ms in relation to fixation onset), for all participants. For the two other algorithms (ADJAR, GLM), the epoch window was defined with a common criterion: it had to be long enough to include at least the temporal description of three evoked responses on adjacent fixations (a current fixation at rank *n*, a previous fixation at rank *n - 1* and a subsequent fixation at rank *η +*1)^7^. Since each participant had his/her own oculomotor pattern, the IFI distributions differed from one participant to another Thus, the epoch window was defined for each participant, (1) to include the contributions of the potentials elicited by the fixation at rank *η - 1*, as well as by that at rank *η +1*, (2) to obtain an almost constant number of fixations inside the epochs and consequently (3) to standardize as far as possible the contributions of the responses that overlap across epochs. For this purpose and for each participant, the epoch window was defined as: [-250-τ;600 +τ] ms, with *τ* being the sum of the individual mean and standard deviation of the IFI values distribution. The average τ value for all participants was equal to 406.37 ms, at around *410* ms^8^. The epoch window was, therefore, on average [-660; 1010] ms, and the number of fixations which occurred within an epoch was on average (std), 5.8 (1.07). The average (std) onset of immediate previous fixations was equal to - 279.51 (22.26) ms, and the average (std) onset of immediate subsequent fixation was equal to 288.43 (24.85) ms.

In the next section, we look at the specificity of the ADJAR algorithm, and of the GLM configuration.

The ADJAR algorithm was applied to the estimate *â^Av^(t)* on the estimation window from -450 ms to 800 ms, and included the interval of interest from -250 to 600 ms. For the iterative procedure, the stopping criterion *C* was defined as the relative power between two consecutive it- erations became lower than a given threshold: 
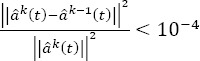
. The estimate by ADJAR, 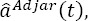 was obtained after 21 (6) iterations on average (std).

For GLM implementation, we worked on the assumption that the same potential was elicited at each fixation onset. We therefore looked at only one class: the *a(t)* po- tential. The onsets of all fixations (not just adjacent ones) during the epochs took into account computation of the Toeplitz *D* matrix, and then estimated the evoked potential. The estimate *â^Glm^(t)* was givenby equation (14). This was applied to each subject and for all electrodes. The dimension of the ***x*** matrix of the observed EEG signals was time-locked at fixation onset (rank *n)* and was *E*×*N_e_* rows and *N_el_* columns. *E* was the number of epochs for a given subject, *N_e_* the number of samples of an epoch (here 1010 + 660+1 = 1671) and *N_el_* the number of electrodes (28). The dimension of the 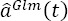 matrix was *N_a_* rows and *N_el_* columns, with *N_a_* the number of samples of the estima- tion window (600+250+1 = 851).

#### Results with the ADJAR algorithm

The ADJAR algorithm was applied to estimate the po- tential elicited at fixation onset during free exploration, since this potential was only overlapped by those elicited by adjacent fixations.

**Figure 1:**
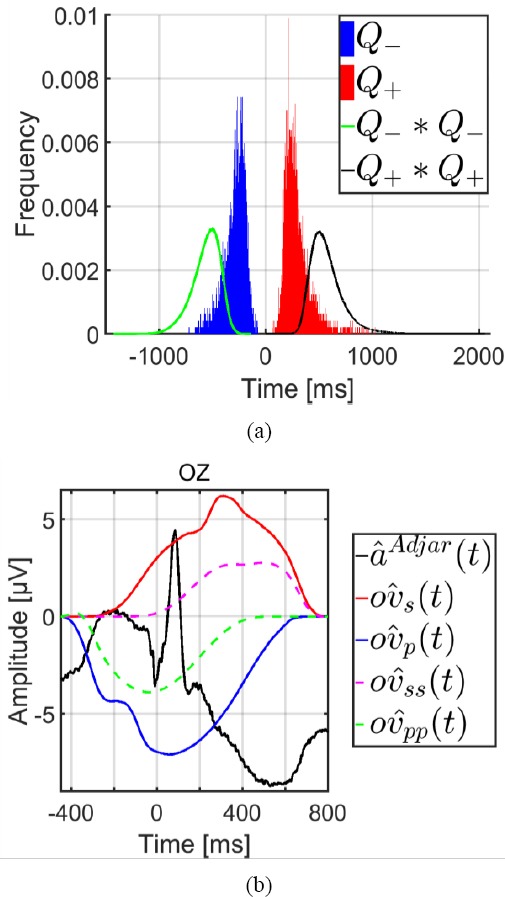
(a) Normalized distributions *Q*_-1_(t), *Q*_+1_(t) and the envelope of these distributions after convolutions: *Q*_-1_(t) * *Q*_-1_(t), *Q*_+1_(t) * *Q*_+1_(t); (b) evoked potential estimated by the ADJAR algorithm 
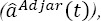
, the first-order 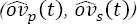 and second-order 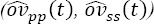 response overlaps on Oz electrode, at convergence

In Figure 1a, normalized distributions of the timestamps of adjacent fixations are plotted for all participants, namely *Q_-1_(t)* and *Q*_+1_(t) and the envelopes^9^ of these distributions are represented after convolution, to give *Q*_-1_(t) * *Q*_-1_(t), and *Q*_+1_(t) * *Q*_+1_(t) The support of these second-order distributions was around [-1000; -150] ms and [270; 1120] ms, respectively, and intersected with the estimation interval. The supports of the resulting higher-order distributions were progressively enlarged, and their contributions decreased progressively as iterations increased.

Figure 1b shows the grand averages after convergence of the algorithm. This figure illustrates the three final estimations given by the algorithm: 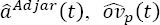 and 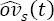. Previous *(0v_pp_(t))* and subsequent (0v_ss_(t)) second-order response overlaps were also estimated^10^ by convolution with the final estimate *â^Adiar^ (t)* and are presented in Figure 1b.

The period [-200; -100] ms before 0 (fixation onset) was used for the baseline correction, In common with other EFRP studies (23), (16), we used the period [-200; -100] ms before 0 (fixation onset) for baseline correction. The saccadic spike potential takes place during the period [-100; 0] ms, due to saccade generation (25). This period therefore has to be excluded from the baseline computation. Finally, the period [-200; -100] ms corresponds to the end of the previous fixation and is free of any activity related to saccade generation.

In Figure 1b, all waveforms are illustrated in the estimation window [-450; 800] ms. Estimations during the first and the last 200 ms were biased by the return to zero of the tapering window. These parts were, as a result, excluded from the [-250; 600] ms window of interest. The contribution of the subsequent second-order response overlap 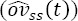 was not corrected in this estimation. The final estimate was under-estimated from 0 ms (positive values for 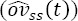). The contribution of the previous second-order response overlap (ov_pp_(t)) was also not corrected. The final estimate was thus over-estimated by up to 400 ms (negative values for ov_pp_(t)), see equation (10). Some of the previous and subsequent second-order response overlaps were not negligible in the estimation window, and contradicted the third assumption of the ADJAR algorithm. The response overlaps were increasingly smoothed by these successive convolution operations, as after a low-pass filtering. However, we must bear in mind that previous and subsequent second-order response over-laps remain in all iterations up to the final convergence.

The baseline (before 0ms) of the waveform *â^Adjar^(t)* presented in Figure 1b, is therefore impacted by the second-order previous response overlap. The return to zero (after 400 ms) was distorted by subsequent second-order response overlaps. Despite these distortions, Figure 1b shows that the potential was mainly composed of the lambda response (latency around 100 ms). The estimated first-order subsequent response overlap (red line; 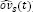), increased between -260 ms and 280 ms, and attained its maximum value maximum between 300 and 370 ms. These latencies were in line with the sum of the lambda component latency and the average onset of the subsequent fixation (≈ 80+288=368 ms). In other words, *ov_s_(t)* had a greater impact on the late components of the evoked potential than on the early ones, here the lambda wave. The large negative deviation from 200 ms observed for *â^Adjar^(t)* was the result of neglecting subsequent second- order response overlaps. The estimated first-order previous overlap response (blue line; 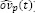), was a negative wave, with a minimum of around 100 ms, i.e. the same latency as the lambda wave. The temporal evolution of this wave was mainly due to the highly negative *â^Adiar^(t)* deviation observed at the end of the window of interest.

Consequently, *ov_p_(t)* had a high influence on the estimation of the lambda wave, and its estimation was highly biased, since previous second-order response overlaps were not considered. Finally, the lambda wave estimation was erroneous.

Therefore, in the context of EFRP estimation, the third hypothesis of the ADJAR algorithm was not validated. The distortions due to second-order response overlaps were not taken into account and were not corrected when estimating the evoked potential, and this resulted in a major bias.

#### Comparison of estimations by average, the ADJAR algorithm and the GLM

**Figure 2:**
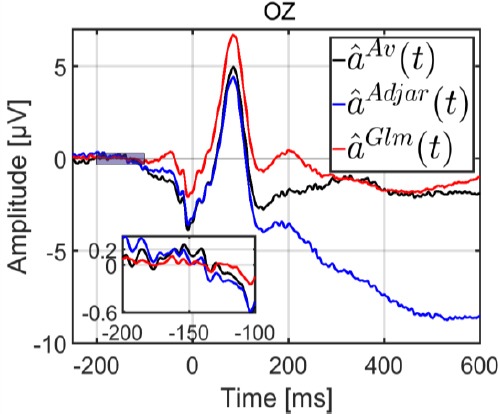
Grand average for the three methods: Average 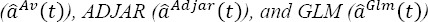 on the OZ electrode; the inset is a zoomed plot between -200 and -100 ms

In this section, we look at the three algorithms used to estimate an EFRP. The grand averages for the three methods are plotted in Figure 2, with a zoomed plot between - 200 and -100 ms. For the Average method, the estimation was performed without taking into account the potentials elicited by previous and subsequent fixations. Only adjacent fixations were considered for the ADJAR estimation, because this algorithm is only able to account for adjacent fixations. The second underlying assumption in the section entitled “Estimation by the ADJAR algorithm” illustrates this. By contrast, the GLM is able to account for all response overlaps. Consequently all fixations inside the epoch were considered for the GLM estimation.

As expected, each method elicited a clearly visible lambda wave (of around 100 ms). The three estimates were evaluated according to two qualitative criteria. The first of these was potential stabilization during the baseline period, and the second was the return to zero of the potential amplitude at the end of the estimation period. In any study of EFRP under experimental conditions, stabilization, i.e. no drift, during the baseline period is needed to obtain a reliable estimation for comparison. We expected a progressive return to zero of the amplitude of the evoked potential at the end of the estimation period. Indeed, neural activities during this late period became less and less synchronized with fixation onset.

Statistical comparison of the variance during the baseline interval, between -200 and -100 ms, revealed that the variance of the estimate *â^Glm^(t)* was lower than the variance of estimates was lower than the variance of estimates Bonferroni adjustment for multiple comparisons). The zoomed plot can be seen in Figure 2. The baseline correction for this period was therefore erroneous for both the ADJAR algorithm and the Average method. As far as the return to zero at the end of the estimation window was concerned, this criterion was only met for the GLM estimate (after 450 ms). Results with a longer estimation window [-400; 1550] ms are presented in the appendix to confirm this statement.

### Estimation by GLM of evoked potentials at the beginning of exploration

Based on the same trials as mentioned previously, the epochs here were time-locked at stimulus onset. We looked at potentials elicited at the beginning of visual exploration: potentials elicited at the first fixation, and the event-related potential at image onset. In this subsection, we discuss the configuration of the GLM depending on the potential of interest. Two GLM configurations are detailed. We then present the condition number of the *D^f^D* matrix as a global indicator of the expected estimation quality. Finally, the results for the different models are presented (according to) potentials of interest.

#### Configuration of selected models

As detailed previously, in order to take into account the response overlap for the estimation of potentials of interest, several GLM configurations can be chosen. These are based on different assumptions.

With a two-class GLM, the potential elicited at each fixation onset was assumed to be the same irrespective of fixation rank. Thus, for a given trial *i*, the observed signal *Xi(t)* at image onset can be written as:


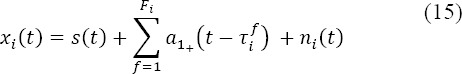


with *S_t_*, the evoked potential at stimulus (image) onset, *a_1+_(t)* the evoked potential at each fixation onset, τ_*i*_^f^, the timestamp of the onset of the *f^th^* fixation rank, occurring in the *i^th^* epoch, and *n*_i_(t) brain activity unrelated to the task. Consequently, for all trials and for each participant, using the same notation as in the section “Estimation by the General Linear Model”, the GLM can be expressed as:


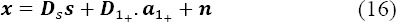


The potentials *s(t)* and *a_1+_(t)* were estimated from equation (14), where 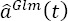 is the vertical concatenation of *ŝ*, and *â_1+_*, horizontal concatenation of *D_s_* and *D_1+_*. See Table 4 (left column).

**Table 4:** Summary of the two GLM configurations

Two-class Model	Three-class model
**x = D_s_S + D_1_+__ a_1_+__ + n**	**x = D_s_S + D_1_a_1_ + D_2_+__a_2_+__ + n**

**S:** Class one, Potential evoked at image onset **a_1_+__:** Class two, EFRP at all fixation onsets at the begin-ning of exploration	**s:** Class one, Potential evoked at image onset **a_1_:** Class two, EFRP at the 1st fixation onset **a_2_+__:** Class three, EFRP atthe 2nd and following fixa-tions

Resolution by equation (14): **â**^Glm^ = (**D^†^D**)^−^1^^ **Dx**	

**x** ∈ ℝ^**N**×**N**^_el_,with N the total number of samples(N = N_e_ × E),E the number of epochs for a given subject, N_e_ the epoch size(N_e_=1500+200+1=1701samples) and N_el_ the number of electrodes (N_el_ = 28)	

**â^Glm^** ∈ ℝ^(N_s_+N_a_)N_el_^**,**with N_s_ = N_e_, N_a_ = 800+200+1=1001samples	**â^Glm^** ∈ ℝ^(N_s_+N_a_)N_el_^**,**with N_s_ = N_e_, N_a_ = 800+200+1=1001samples

**D** = [D_s_,D_1_+__], with D ∈ ℝ^N×(N_s_+N_a_)^	**D** = [D_s_,D_1_,D_2_+__], with D ∈ ℝ^N×(N_s_+2.N_a_)^

*Overall estimation of quality: condition number of the***D**†**D***matrix*

However, analysis of statistical behavior (see section entitled “Behavioral results”) revealed significant differences between the first fixation -in terms of duration, incoming saccade amplitude, contrast of the gazed foveal region- and subsequent fixations. Part of the EFRP is explained by the lambda potential which reflects the visual change in the image retina due to the saccade (3) (18); (37). At fixation onset, the lambda potential depends not only on low level image features (luminance and contrast of the area gazed at), but also on the incoming saccade amplitude (41), (31), (37). As we were aware of these modulations early in the case of EFRP, we were able to consider an alternative GLM as a supplementary class for the first EFRP. Thus, equation (3) can be rewritten as:


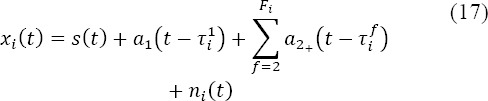


with *a_1_(t)* being the potential elicited specifically at the onset of the first fixation, and *a_2+_(t)*, the potential elicited at the onset of the second and subsequent fixations. The equation of the corresponding GLM is then:


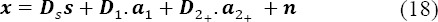


The potentials *s(t)*, *a_1_(t)*, and *a_2+_(t)* were estimated by equation (14), where *â^Glm^* is the vertical concatenation of *ŝ*, *â_1_* and *â_2+_* and *D* is the horizontal concatenation of ***D***_s_, ***D***_1_, and ****D***_2+_*. See Table 4 (right column).

All results were obtained using the following configurations for the epochs:

- We chose an epoch duration which was long enough to include at least the temporal description of the first two evoked responses on adjacent fixations (the first and second ones). This epoch was defined as follows: [-200; 800 + τ] ms, with τ being the sum of the average of the latency of the second fixation (561.50 ms), and its standard deviation (80.85 ms): τ = 561.50 + 80.85 = 642.35 ≈ 700 ms. See Table 2. Consequently for all trials and participants, the temporal interval of epochs was [-200 ; 1500] ms from image onset. On average (std), the number of fixation onsets in an epoch was 5 (1). The number of epochs per subject was equal to the number of trials.
- The duration of the estimation window for the evoked potential (*s(t)*) at image onset was the same as that of the epoch.
- The estimation window for the evoked potential at fixation onset *(a_1_(t), a_1+_(t), a_2+_(t))* was from -200 ms to 800 ms.

Using the GLM, potentials were estimated by equation (14). This required computation of the inverse of the***D**†**D*** matrix. For the two-class GLM, the *D* matrix was composed as *D =* [D_S_,D_1+_] (equation (16)), and for the three-class GLM, the *D* matrix was composed as *D = [D_s_,D_}_,D2_+_]* (equation (18)). The***D**†**D*** matrix is a block matrix: a 2 x 2 block matrix for the two-class GLM and a 3 x 3 block matrix for the three-class GLM.

In general, the condition number of the ***D**†**D*** matrix is taken as an indicator (or a warning) of an estimation’s re- liability. With a greater condition number, there is a risk that the noise *n(t)* on observed data might be amplified on the solutions *a(t)* of the mean square error problem (equation 13). More specifically, on average, the variance of the estimate (expressed in the appendix) depends on the inverse of ***D***^†^***D*** and thus on its condition number.

A bootstrap estimator with 10 000 replications was implemented for statistical assessment of the condition number of the ***D***^†^***D*** matrix for each model, and each participant.

On average (std), the condition number (CN) for the three-class model *(CN = 1446.70 (834.36))* was higher than that of the two-class model *(CN = 707.36 (322.26))*, and much higher than that of the model by average (*CN =* 1)^11^. The first class gathered together events at image onset and the associated timestamps all equaled zero. Therefore, for the two-class model, the jitter on the timestamps between the two classes was accurately represented by the variability of the timestamps of events within the second class. In other words, the condition number for this model was directly linked to the variability of all fixation onsets, irrespective of their rank. In the case of the model with three classes, this set of fixation onsets was split into two parts. The first fixation (second class) was separated from subsequent ones (third class). The timestamp variability for the first fixation onset was therefore smaller than that of the following fixation onsets. It was for this reason that on average, the condition number for the two-class model was better (lower value) than for the three-class model.

**Figure 3:**
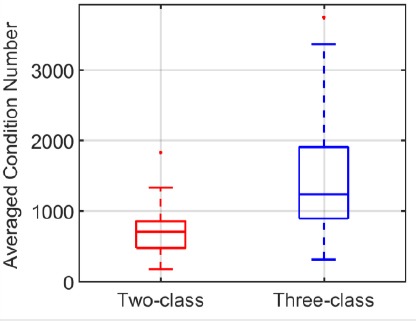
Averaged condition number of the ***D***^†^***D*** matrix for the two GLM configurations, with two or three classes, based on individual means computed with a bootstrap estimator.

Figure 3^12^ illustrated the distribution of condition numbers based on individual means obtained by bootstrap estimation. Because of the link, on average, between the condition number and the estimation variance, the result concerning the condition number of the ***D***^†^***D*** matrix for each model ought to predict a larger variance for the three- class than for the two-class model and for the model established by averaging (one-class model). In view of the tradeoff between bias and variance, this decrease should be associated with an increase in estimation bias. In practice, since a high-level condition number was only a warning, the bootstrap estimator of the variance of evoked potentials was systematically computed in order to obtain a quantitative criterion for the assessment of estimates.

In the next two parts, we study the two GLM configurations in order to estimate different potentials of interest. In the subsection “EFRP at first fixation onset”, the potential of interest is the EFRP at the first fixation onset. The three-class model is used; the EFRP at the first fixation onset is compared to EFRPs at subsequent onsets, in order to evaluate the specificity of the first fixation. In the following subsection “Evoked potential at image onset” the average and the two GLM configurations are compared to assess the impact of the first EFRP estimation on the estimation of the ERP at image onset.

Table 5 summarizes the content of the two following subsections.

**Table 5:** Summary of model choices related to the potential of interest for the two following subsections

Subsection name	Potential of interest	Estimation method
EFRP at first fixation onset	a_1_(t)	Three-class model

Evoked potential at ima2e onset	s(t)	i.Average ii.Two-class model iii.Three-class model

#### EFRP at first fixation onset

We considered the EFRP estimation at the first fixation onset. We showed that ocular features (incoming saccade amplitude, fixation duration) and the local luminance contrast of the foveal region gazed at were different for the first fixation than for the following ones (see above in section “Behavioral results”). This justifies establishing a specific class to estimate the first EFRP, and is in line with methodological studies (15), (29).

In the case of the three-class GLM (Table 4, right column), the potential of interest *a*_1_(*t*) was associated with the second class. The first and third classes provided an unbiased estimation of the EFRP at the first fixation by taking into account the influence of the ERP *s(t)* at image onset as well as overlaps of the response *a_2+_(t)* at subsequent fixations.

**Figure 4:**
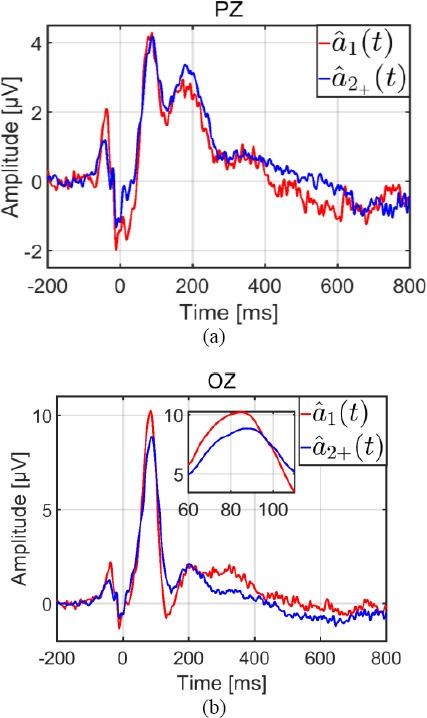
Grand average of the EFRP at the first fixation *â_1_(t)* (red line) and at the second and subsequent fixations *â_2+_(t)* (blue line), estimated by the three-class GLM (a) on PZ and (b) OZ electrodes

Using the three-class GLM, Figure 4 illustrates the estimation of the first EFRP *â^t)*, and of subsequent ones *â_2+_(t)*, on PZ and OZ electrodes. In line with common practice, these estimates were corrected with a baseline of between -200 and -100 ms.

**Figure 5:**
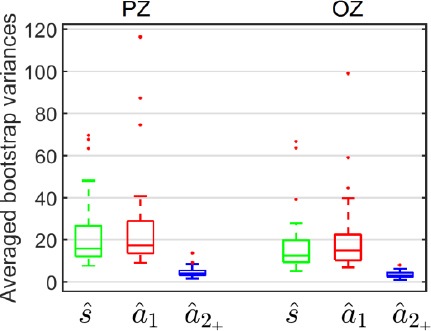
Averaged bootstrap variances of the ERP at image onset *ŝ(t)*, the EFRP at the first fixation *â_1_(t)* and at the second and subsequent fixations *â_2+_(t)*, estimated by the three- class GLM, on PZ and OZ electrodes

The component of interest was the lambda response between 70 ms and 90 ms. Inside this temporal interval, the difference between was not significant on the PZ electrode according to a t-test: *t(33) = 0.49, ρ = 0.63*. However, on the OZ electrode, the same difference was significant: *t(33) = 2.69, ρ =* 0.01. This result confirmed the assumption that the first fixation would have to be differentiated from following ones. As Figure 5 shows, the variances of both estimates were also different, that of *â_1_(t)* being larger than *â_2+_ (t)*.

The bootstrap variances were statistically analyzed using a repeated measures ANOVA with the class(*ŝ*(t), *â_1_(t)*), *â_2+_(t))* and the electrode (PZ, OZ) as within-partic- ipant factors. Multiple comparisons were assessed with Bonferroni post-hoc tests. The statistical results revealed significant differences according to class *(F(2,66) = 36.62, ρ < 0.001)*, to electrodes *(F(1,33) = 10.71, ρ =* 0.002) and to both *(F(2,66) = 751, ρ =* 0.001). In the light of the main effect on electrodes, the variance of the estimate *a_2+_(t)* was lower than the variances of the estimates s(*t) and â_1_(t)* which were similar. These results were expected. Firstly, *â_2+_(t)* was estimated from more samples (on average four fixations with a rank greater than one per epoch), than *â^t)* (a single first fixation per epoch). Secondly, *s (t)* and *â_1_(t)* were estimated from only one event per epoch, for each potential.

#### Evoked potential at image onset

The potential of interest was the potential elicited at image onset, i.e. the *s(t)* waveform, or the *s* vector, in equations (16) and (18). After image display, the onset of the first fixation occurred just after the first saccade, on average (std) at 307.65 (46.14) ms (cf. Table 2). The potentials elicited by this ocular event and by subsequent ones, provided distortions by overlapping on this potential of interest.

For comparison *ŝ^(0)^*(*t*) expresses the ERP estimated by the average, *ŝ^(1)^ (t)* and *ŝ^(2)^(t)* the ERP estimated by the GLM with two (equation (16)) and three (equation (18)) classes, respectively.

**Figure 6:**
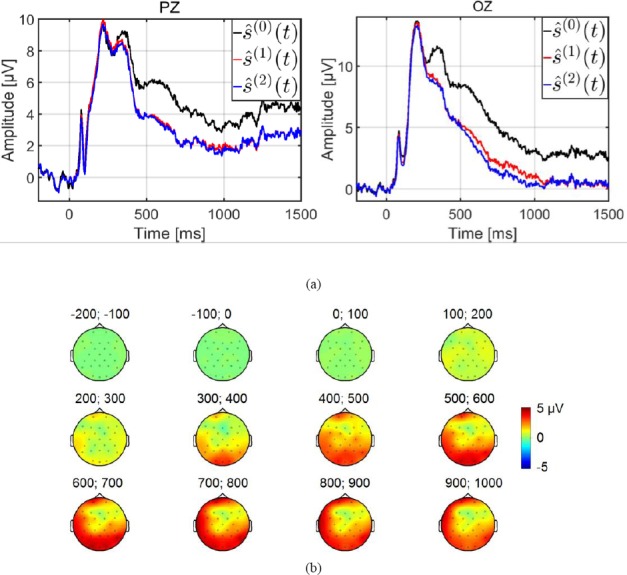
(a) Evoked potential at image onset on the midline PZ and OZ electrodes for the three estimations, by average *ŝ^0^(t)*, by the two-class GLM *ŝ^1^(t)* and by the three-class GLM *ŝ^2^(t)* (b) Topographic maps of the difference between the two estimates: *ŝ^0^(t)*-*ŝ^1^(t)*

Figure 6a illustrates the estimations of the potential evoked at image onset by the three methods, after a base line correction on OZ and PZ electrodes from -200 to 0 ms. Firstly, early and late components were observed for up to 600 ms before a return to a stabilized level. Interestingly, this stabilized level at the end of the segment was highest for the classical estimation *ŝ*^(0)^_(*t*)_, showing that residual activities from all potentials elicited at fixation onsets provided on average a positive bias.

Irrespective of the estimation method or choice of model, the P1 early component was clearly identifiable (Figure 6a), with a similar pattern (shape, maximum, latency). In other words, the estimation of the P1 component was not affected by distortions from overlaps. This was as expected, because the latency of this component (around 90 ms) was shorter than the latency of the first saccade (on average (std) 266.85 (44.57) ms, see Table 2. This also explains why the estimation of the P2 component from the latency around 200 ms differed from one method of estimation to another (Average vs GLM).

The large gap between ŝ(0)(*t*) and ŝ(1)(*t*) (or between ŝ(0)(*t*) and ŝ(2)(*t*) occurred from 300 ms on, and a higher maximum for s(0)(*t)* was observed at around 360 ms. The interpretation of these distortions on ŝ(0)(*t*) was derived from the contribution of the potential elicited by the first fixation onset. On average, across all trials, this contribution corresponded to the convolution of the first evoked potential *a_}_(t)* with the distribution of Dirac impulses at the timestamps of the first fixation onset. The average (std) of this distribution among participants was 307.65 (46.14) ms (see Table 2). The result of this convolution was a low- pass version of the first evoked potential *a_}_(t)*, with a lambda wave peaking at around 90 ms, then shifted at the average onset of the first fixation. This explained why aless steep maximum was observed on ŝ(0)(*t*) at a latency around 90 +307.65 ≈390 ms

Figure 6b shows the topographic map of the difference between ŝ(0)(*t*) and ŝ(1)(*t*)**. The positive gap between ŝ(0)(*t*) and ŝ(1)(*t*)*)* started at 300 ms in the occipito-parietal area, and went on to progressively cover the whole scalp almost uniformly, with a gap value of a few microvolts (near 2 μν, on PZ and OZ electrodes). In other words, from the ŝ(0)(*t*) estimate, the analysis of components with latencies above 300 ms yielded erroneous interpretations. In contrast, the period ranging from image onset to the first saccade (on average 266.85 ms, see Table 2), was free of eye movement.

Consequently, the estimations obtained by simple averaging and by the GLM were similar, as observed for instance for the early P1 component.

The selection of each of the three models (Table 5) was analyzed in terms of bias and variance of the estimation. Estimation by average (ŝ(0)(*t*)*)* could be expected to provide the most biased estimator of the three. In contrast, by making a distinction between the first EFRP *t*)) and the following EFRP (*a_2+_(t))*, the three-class GLM could be expected to provide the estimate with the lowest bias.

**Figure 7:**
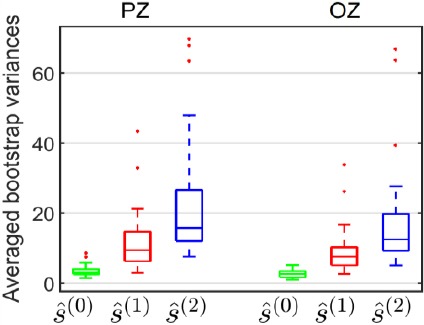
Averaged bootstrap variances for the three estimations of the evoked potential at stimulus onset, on PZ and OZ electrodes: by average *ŝ^0^(t)*, by the two-class GLM *ŝ^1^(t)* and by the three-class GLM *ŝ^2^(t)*

Moreover, no significant difference was observed between and *s(^1^)(t)* and ŝ(2)(*t*) estimates (Figure 6as); a similar bias for these two estimates was assumed. However, the assumption for the variance was that the variances for the three-class model would be greater than the variances of the two-class model, because the former is less parsimonious than the latter. These variances were evaluated using 10 000 bootstrap repetitions for each model, each electrode and each participant. Figure 7 shows the bootstrap variances averaged for participants, on PZ and OZ electrodes, for the three estimates of the evoked potential at image onset. These bootstrap variances were statistically analyzed using a repeated measures ANOVA with model choice (Average for ŝ(0)(*t*),**, two-class GLM for s^t), three-class GLM for s(^1^)(*t))* and electrode (PZ, OZ) as within-participant factors. Multiple comparisons were assessed with Bonferroni post-hoc tests. The statistical results revealed significant differences according to the model *(F(2,66) = 45.47, ρ < 0.001)*, to the electrodes (F(1,33) = 8.09, *ρ = 0.007)* and to both *(F(2,66) =* 5.85, *ρ =* 0.005). As expected, on both electrodes, the variance of the estimate by the three-class GLM (ŝ(2)(*t*)) was greater than the variance of the estimate by the two-class GLM (ŝ(1)(*t*)) which was in return greater than that of the estimate obtained by the average (ŝ(0)(*t*))

These results were in agreement with the increased number of parameters for the first, second and third model relative to a fixed number of observed data, and were consistent with the increasing profile of the condition number of the ***D***^†^***D*** matrix for the three models (Figure 3).

## Discussion

One of the main limitations of EFRP estimation is the overlapping issue between adjacent neural responses. This is due to IFI values which are too low (15); (29). The amount of overlapping is linked to the ocular sequences of each participant, and is not controlled by experimental design, except in specific cases (24). To address this difficulty, Kaunitz and colleagues (24) trained participants to make long fixations. All other things being equal, this increases IFI values and consequently decreases overlap between adjacent potentials.

It has been well-established that the estimation of evoked potentials by averaging time-locked EEG signals is biased in the case of overlapping responses. Woldorff (40) proposed an iterative procedure in the context of ERP experiments where the EEG signal is time- locked on external events. This was called the ADJAR algorithm, and was designed to estimate overlap responses from immediately adjacent events, to converge towards the evoked potential of interest. Moreover, regression techsniques, especially the GLM (26), have proved their efficiency in the estimation of evoked overlapping potentials (8); (9); (6); (2); (27). The ADJAR algorithm and the GLM are applied to experimental data from conjoint EEG and eye- tracking recordings during free visual exploration, and are compared on the basis of their efficiency in relation to overlap responses for the estimation of evoked potentials.

Response overlaps are added linearly to the potential elicited at the time-locked event in both models. Only temporal delays are considered in the estimation of previous or subsequent response overlaps. In the case of such comparisons between the ADJAR algorithm, and the GLM, the same type of evoked responses for all events is considered. This restriction can be removed in some cases for the AD- JAR algorithm (35). However, taking into account various types of evoked potentials during trials is easy when using the GLM. This comparison therefore focuses on overlaps coming from the same type of potential

In the ADJAR model, only adjacent responses (immediately previous and subsequent fixations) are considered. Thus, for this model, the potential of interest is *assumed* to be overlapped only by potentials elicited by the previous or subsequent event. This strong assumption is at the core of the definition of the ADJAR iterative process. The AD- JAR algorithm is based on the definition of the normalized distribution of the timestamps of the fixation onsets of previous and subsequent fixations. This means that the contribution of all events inside each epoch must be the same, and this is a necessary condition of convergence. For this reason *only* two adjacent fixations (the previous and the subsequent one) are taken into account in the epoch. We have shown that this assumption fails in the context of the EFRP estimation. The window of interest is chosen to include early and late components of the evoked potential. This potential is estimated within an estimation window which is slightly larger than the window of interest, and which includes extra time to accommodate the border effects of convolution products. In practice, this extra time is of the same magnitude as the IFI value. Consequently, more than two evoked potentials on fixations overlap the EFRP of interest. Moreover, two main issues can lead to a non-negligible contribution of second-order adjacent response overlaps at convergence: an insufficiently long IFI and a low variability. At the last iteration, the estimated potential remains biased by these second-order responses, for which overlaps are not corrected. To sum up, the AD- JAR algorithm appears to be poorly suited to EFRP estimation.

The comparison with the GLM shows a more natural framework in this context. All events can be taken into account inside each epoch without restriction by a closed- form estimation: there is no iterative procedure, and therefore no convergence issue, and no stopping criterion needs to be defined.

In addition, the ability of the GLM to deconvolve different neural responses is illustrated via two situations depending on the potential of interest: the potential elicited (1) at the first fixation onset and (2) at image onset. To this end, we considered two GLM configurations, with two and three classes. For the first model, events were split into two classes: image onset and fixation onsets irrespective of fixation rank. For the second model, the events were split into three classes: image onset, the first fixation onset and the following fixation onsets. The two-class model assumes that the same potential was elicited at fixation onset irrespective of fixation rank. In contrast, the three-class model establishes a distinction between the potential elicited by the first fixation and by subsequent ones, and assumes a specific status or particular features for the first fixation at the beginning of the task. The choice between these two models depends not only on the issue in visual perception, or reading, but also on the quality of estimations. If the potential of interest is the first EFRP, the three- class model is fully justified. If the potential of interest is the one elicited at stimulus onset both models can be used. The choice should be based on additional criteria.

The three-class model is suitable if the potential of interest is the one elicited at the first fixation onset. The estimated potentials corresponding to the first and third class, provide an unbiased estimation of the potential of interest (corresponding to the second class). However, the variances of the estimation of the first EFRP and of the ERP at image onset are of the same magnitude. Moreover, these two variances are greater than the variance of the estimated EFRP at subsequent fixation onsets. This latter potential is estimated using more data (on average 3.9 fixations with a rank greater than one, inside each epoch) than the two former ones (only one event each inside each epoch). The results obtained show that the first fixation differs from the following ones by its significantly shorter duration and by the smaller amplitude of its incoming saccade. The local luminance contrast of the regions gazed at during the first fixation is also significantly higher than for regions gazed at later. With the three-class GLM, the estimated potential elicited at the first fixation onset exhibits a significantly larger amplitude of the lambda wave than the amplitude of this wave elicited by subsequent fixations. Modulation of the lambda wave amplitude by incoming saccade amplitude has been well-established (41), (37), (31), (24), (29). The greater the incoming saccade amplitude is, the larger the lambda amplitude will be. The same holds true for the physical properties of visual stimuli such as illuminance (34), (18) (44). A smaller first incoming saccade amplitude implies a decrease in the amplitude of the lambda component for the first estimated EFRP. A greater local contrast of the first region gazed upon is observed, but no significant difference is observed for local luminance. Taken together, the observation of a greater first lambda amplitude cannot be explained by these low- level features. High level factors such as task demand and information processing load also modulate the lambda amplitude (42); (32). A higher level of attention may be assumed at the beginning of a task. This could be one interpretation of the greater amplitude of the lambda wave elicited at the first fixation onset, following the same speculations in (42); (43).

The two-class or the three-class model can be designed to estimate the potential elicited at image onset. There is no definitive result on this choice, which depends on the classical tradeoff between bias and variance. A priori, the distinction between the first EFRP and the following ones makes sense based on ocular behavior. This distinction was ignored in the two-class GLM. There is therefore a risk that a biased estimation of the EFRP irrespective of fixation rank, and consequently of the ERP at image onset, might occur because of a poor fit between the model and the observed data. The more distinctive first EFRP is, the greater the level of risk becomes. This risk of a biased estimation is balanced out by a lower variance. In our study, the difference between the estimates for the ERP at image onset given by the two models was not significant, but the variance for the three-class model was higher. This result justifies the choice of the two-class model for the estimation of the potential elicited at image onset.

In the context of EFRP, the GLM is a useful model to estimate overlapped evoked potentials. The GLM also allows the estimation of different neural responses. The main assumption is the linearity of the additive model to take into account different neural responses. The configuration of the number of classes depends on the assumptions concerning the cognitive processes under examination. As far as estimation is concerned, the number of classes results from the classical tradeoff between bias and variance. In order to obtain the best configuration of the GLM, two main questions must be asked: “what is the potential of interest?”, and “what are the related potentials which may affect the estimation of the potential of interest?”. The answer to the first question is often trivial. However, the answer to the second one is not. This is the outcome of a tradeoff between the parsimony level of the GLM and the quality of estimation. The higher the class number is, the more accurate and less parsimonious the model becomes, and the more the quality of the estimation is affected. Guidelines are presented to help with model selection: the condition number and the estimation variances must be evaluated in relation to the jitter between the timestamps of events. Sufficient jitter on the timestamps within and between classes is necessary to prevent near collinearity in the ***D***^†^***D*** matrix. In other words, sufficient jitter is essential to provide reliable estimates (2), i.e. to be able to separate the overlapped responses. Estimation of the potentials requires the inversion of the ***D***^†^***D*** matrix. The overall quality can be evaluated by the condition number of this matrix. For a given model, greater jitter allows the condition number to be reduced. Consequently the quality of the estimation should be increased by ensuring better separation between the overlapped potentials. From the point of view of model selection, increasing the number of classes is often combined with a decrease in in- tra-class jitter. Consequently, the condition number should increase, along with the variance estimation. Choosing the model with the highest number of classes means that a lower bias is favored over variance increase. For this reason, we suggest that this variance should be estimated in order to allow informed model selection.

The GLM is a very popular method and has been proposed in numerous studies as a meaningful tool to linearly deconvolve overlapped responses (28); (33); (10); (6); (14); (2), (Smith & Kutas, 2015ab); (7). However, in the context of EFRP, this methodology is not yet widely used (9); (14); (13), while the overlapping issue remains a major concern. Moreover, in some cases, the assumptions of linear additive mixing of time-invariant responses may appear limited for the estimation of auditory evoked potentials, as discussed in (2). Appropriate nonlinear models need to be designed to overcome these limitations. Nevertheless, the Generalized Additive Mixed-Effects Model (GAMM) has recently been proposed to take into account nonlinear relationships between co-variables where necessary (38). Both the GLM, and the GAMM, are powerful statistical models for the EFRP estimation in complex situations with overlaps and modulations through both low-level (oculomotor behavior, stimulus properties) and high-level features (such as attentional resources, and arousal).
