# Supplementary material for: Estimation of overlapped Eye Fixation Related Potentials: The General Linear Model, a more flexible framework than the ADJAR algorithm
Source: J Eye Mov Res. 2017 Oct 7;10(1):10.16910/jemr.10.1.7. doi: 10.16910/jemr.10.1.7 (PMC7141057; doi:10.16910/jemr.10.1.7)
Supplement: Supplementary file 1 [file JEMR-10-1_appendix.pdf]

Appendix

This appendix gathers together a number technical comments concerning the ADJAR algorithm, and details of the full expressions of the second and third iterations. Finally, it features the theoretical expression of the variance for GLM estimation.

Description of ADJAR algorithm implementation

The ADJAR computation is based on the distributions $Q_{+1}(t)$ and $Q_{+1}(t)$ that are extracted from the set of epochs. The ADJAR algorithm for each iteration is based on a succession of convolution products. Theoretically, all convolutions used in the ADJAR algorithm involve signals defined from minus infinity to plus infinity. However, in practice, a finite number of samples had to be dealt with. To prevent the border effects of convolution and of signal truncation, the estimation window for the evoked potential is chosen to be larger than its window of interest, and a tapering window $h(t)$ is applied after each convolution throughout the estimation window. In our case, the window of interest is equal to $[T_o; T]$ ($T_o = -250$ ms and $T = 600$ ms) and the larger estimation window is equal to $[T_{start}; T_{end}]$ ($T_{start} = -450$ ms and $T_{end} = 800$ ms). Figure 8a illustrates these windows. For the tapering window $h(t)$, a Tukey window with a coefficient of 0.32 was chosen, instead of the usual Hamming window. The Tukey and Hamming windows are plotted on the estimation window in Figure 9. The Tukey window preserves the signal between -250 and 600 ms (the amplitude is equal to 1), inside the window of interest, unlike the Hamming window.

The convolutions of the waveform by the distributions shift the waveform to the left (convolution by Q_{-1}(t)) or to the right (convolution by Q_{+1}(t)). Figure 8b illustrates the result of the convolution with the distribution Q_{+1}(t), which is shifted to the right. We consider the signal in the estimation window from T_{start} to T_{end} and the distribution Q_{+1}(t) with a support between $T_{Q_{start}}$ and $T_{Q_{end}}$. The signal after convolution is shifted to the right and its temporal support is larger between $T_{C_1} = T_{Q_{start}} + T_{start}$ and $T_{C_2} = T_{Q_{end}} + T_{end}$. Then, after each convolution, the result is truncated with the tapering window to take into consideration only the signal that remained in the estimation window. The steps at each iteration are defined here:

$$\widehat{ov}_s^k(t) = Q_{+1}(t) * \left(\hat{a}^{Av}(t) - \widehat{ov}_p^{k-1}(t)\right) \qquad (19)$$

$$\widehat{ov}_s^k(t) \leftarrow h(t).\widehat{ov}_s^k(t) \qquad (20)$$

$$\widehat{ov}_p^k(t) = Q_{-1}(t) * \left(\hat{a}^{Av}(t) - \widehat{ov}_s^k(t)\right) \qquad (21)$$

$$\widehat{ov}_p^k(t) \leftarrow h(t).\widehat{ov}_p^k(t) \qquad (22)$$

where $h(t)$ is a tapering window along the estimation window $[T_{start}; T_{end}]$.

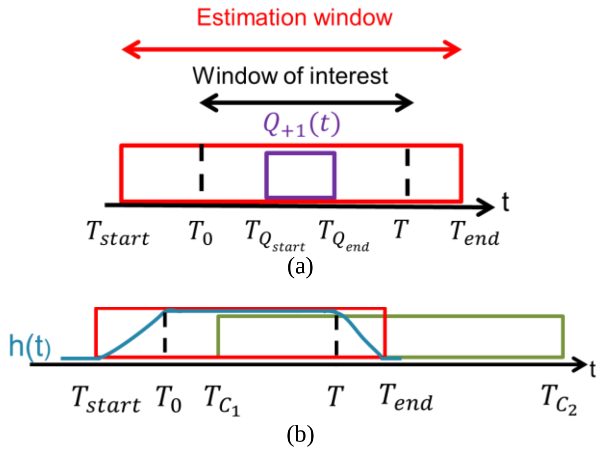

Figure 8 Illustrations of a) the estimation window and the window of interest b) temporal support of the output signal by the convolution with the distribution $Q_{+1}(t)$, between T_{C_1} and T_{C_2}. The tapering window h(t) is in blue

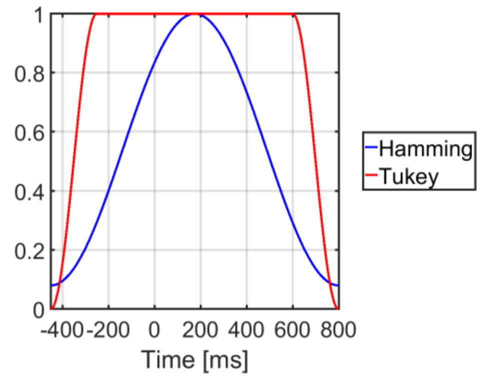

Figure 9 Tukey and Hamming windows

Development of the iterative procedure for the ADJAR algorithm

Adjacent response overlaps ($ov_p(t)$ and $ov_s(t)$) are estimated by an iterative procedure. At convergence, they are subtracted from the evoked potential estimated by the av-

erage of the time-locked signal $\hat{a}^{Av}(t)$ to obtain an estimation $\hat{a}^{Adjar}(t)$ of the underlying potential $a(t)$. The model is:

$$\hat{a}^{Av}(t) = a(t) + ov_p(t) + ov_s(t), \tag{23}$$

$$ov_p(t) = Q_{-1}(t) * a(t),$$

$$ov_s(t) = Q_{+1}(t) * a(t),$$

where $Q_{-1}(t)$, respectively $Q_{+1}(t)$, is the normalized distribution of the timestamps at the onset of the previous and subsequent fixations respectively. Starting from $\widehat{ov}_p^0(t) = 0$, equations (7) and (8) in the main part of the article, describe the kth estimation of the response overlaps. We describe here the development of the first interactions in order to show in the final estimation, the particular status of second-order response overlaps highlighted in red in the following equations.

At the first iteration:

$$\widehat{ov}_s^1(t) = Q_{+1}(t) * \hat{a}^{Av}(t) \tag{24}$$

$$\widehat{ov}_s^1(t) = Q_{+1}(t) * a(t) + Q_{+1}(t) * Q_{+1}(t) * a(t) + Q_{+1}(t) * Q_{-1}(t) * a(t)$$

$$\widehat{ov}_p^1(t) = Q_{-1}(t) * \left(\hat{a}^{Av}(t) - \widehat{ov}_s^1(t)\right) \tag{25}$$

$$\widehat{ov}_p^1(t) = Q_{-1}(t) * a(t) + Q_{-1}(t) * Q_{-1}(t) * a(t) - Q_{-1}(t) * Q_{+1}(t) * Q_{+1}(t) * a(t) - Q_{-1}(t) * Q_{+1}(t) * Q_{-1}(t) * a(t)$$

$$\hat{a}^1(t) = \hat{a}^{Av}(t) - \widehat{ov}_s^1(t) - \widehat{ov}_p^1(t) \tag{26}$$

$$\hat{a}^1(t) = a(t) - Q_{+1}(t) * Q_{+1}(t) * a(t) - Q_{-1}(t) * Q_{-1}(t) * a(t) - Q_{+1}(t) * Q_{-1}(t) * a(t) + Q(t) * Q_{+1}(t) * Q_{+1}(t) * a(t) + Q_{-1}(t) * Q_{+1}(t) * Q_{-1}(t) * a(t)$$

At the second iteration:

$$\widehat{ov}_s^2(t) = Q_{+1}(t) * \left(\hat{a}^{Av}(t) - \widehat{ov}_p^1(t)\right) \tag{27}$$

$$\widehat{ov}_s^2(t) = Q_{+1}(t) * a(t) + Q_{+1}(t)^{(*2)} * a(t) - Q_{+1}(t) * Q_{-1}(t)^{(*2)} * a(t) + Q_{-1}(t) * Q_{+1}(t)^{(*3)} * a(t) + Q_{+1}(t)^{(*2)} * Q_{-1}(t)^{(*2)} * a(t)$$

$$\widehat{ov}_p^2(t) = Q_{-1}(t) * \left(\hat{a}^{Av}(t) - \widehat{ov}_s^2(t)\right) \tag{28}$$

$$\widehat{ov}_p^2(t) = Q_{-1}(t) * a(t) + Q_{-1}(t)^{(*2)} * a(t) - Q_{-1}(t) * Q_{+1}(t)^{(*2)} * a(t) + Q_{+1}(t) * Q_{-1}(t)^{(*3)} * a(t) - Q_{-1}(t)^{(*3)} * Q_{+1}(t)^{(*3)} * a(t) - Q_{+1}(t)^{(*2)} * Q_{-1}(t)^{(*3)} * a(t)$$

$$\hat{a}^2(t) = \hat{a}^{Av}(t) - \widehat{ov}_s^2(t) - \widehat{ov}_p^2(t) \tag{29}$$

$$\hat{a}^2(t) = a(t) - Q_{+1}(t)^{(*2)} * a(t) - Q_{-1}(t)^{(*2)} * a(t) + Q_{+1}(t) * Q_{-1}(t)^{(*2)} * a(t) + Q_{-1}(t) * Q_{+1}(t)^{(*2)} * a(t) - Q_{-1}(t) * Q_{+1}(t)^{(*3)} * a(t) - Q_{+1}(t) * Q_{-1}(t)^{(*3)} * a(t) - Q_{+1}(t)^{(*2)} * Q_{-1}(t)^{(*2)} * a(t) + Q_{-1}(t)^{(*3)} * Q_{+1}(t)^{(*3)} * a(t) + Q_{+1}(t)^{(*2)} * Q_{-1}(t)^{(*3)} * a(t)$$

And so on. For any k^{th} iteration, the terms $Q_{+1}(t)^{(*2)} * a(t)$ and $Q_{-1}(t)^{(*2)} * a(t)$ remain in the estimate $\hat{a}^k(t)$. The other high-order terms are progressively shifted outside the estimation window, due to the combination of $Q_{-1}(t)$ and $Q_{+1}(t)$. At convergence, the final estimations of the response overlaps are:

$$\widehat{ov}_p^{\infty}(t) = Q_{-1}(t) * a(t) + Q_{-1}(t)^{(*2)} * a(t) +/- \sum high\ order\ ov_p \tag{30}$$

$$\widehat{ov}_s^{\infty}(t) = Q_{+1}(t) * a(t) + Q_{+1}(t)^{(*2)} * a(t) +/- \sum high\ order\ ov_s \tag{31}$$

At convergence, the final estimate $\hat{a}^{Adjar}(t)$ can be expressed as:

$$\hat{a}^{Adjar}(t) = \hat{a}^{Av}(t) - \widehat{ov}_s^{\infty}(t) - \widehat{ov}_p^{\infty}(t)$$

$$\hat{a}^{Adjar}(t) = a(t) - Q_{-1}(t)^{(*2)} * a(t) - Q_{+1}(t)^{(*2)} * a(t) +/- \sum high\ order\ overlaps \tag{32}$$

This last equation corresponds to equation (10) in the main part of the article.

Convergence issue of a modified ADJAR algorithm

It is tempting to adapt the ADJAR algorithm to take into account all previous and subsequent fixations, as they effectively occur during the epoch. Indeed, the basic principle of the ADJAR algorithm is to estimate the evoked

potential at the fixation of interest by estimating the overlaps of previous and subsequent responses, taking into consideration only three evoked potentials which occur during an epoch. However in practice, more than three fixations (the fixation of interest, the previous and the subsequent ones) can occur during a single epoch. Consequently, equation (23) is rewritten as:

$$\hat{a}^{Av}(t) = a(t) + Q_{-}(t) * a(t) + Q_{+}(t) * a(t) \qquad (33)$$

$$Q_{-}(t) = \sum_{f=-1}^{-F_p} Q_f(t) = \frac{1}{E}\sum_{i=1}^{E}\sum_{f=-1}^{-F_p} \delta(t-\tau_i^f)$$

$$Q_{+}(t) = \sum_{f=1}^{F_s} Q_f(t) = \frac{1}{E}\sum_{i=1}^{E}\sum_{f=1}^{F_s} \delta(t-\tau_i^f)$$

where $Q_{-}(t)$ and $Q_{+}(t)$ are the distributions of all previous and subsequent fixation onsets across all epochs, respectively. Since the number of fixations per epoch can be greater than three (previous, current and subsequent) in this case, these distributions cannot be normalized to one (i.e. their integral can differ from one). In one epoch, there is one current fixation (the EEG signal is time-locked on this fixation onset), and possibly more than one previous fixation, and also more than one subsequent fixation. The integral of $Q_{-}(t)$ (resp. $Q_{+}(t)$) 's elements is equal to the average number of previous (resp. subsequent) fixations occurring in the epochs. This is possibly greater than the number of the epochs, E. By dividing by E, the sum of the contribution of $Q_{+}(t)$'s elements (or $Q_{-}(t)$) can be greater than one. In order to take into account all fixations during an epoch, $Q_{-1}(t)$ (resp. $Q_{+1}(t)$) is replaced by $Q_{-}(t)$ (resp. $Q_{+}(t)$) in equations (24) and (25) to estimate the current EFRP. This modified ADJAR algorithm was implemented to estimate the EFRP in the middle of visual exploration. During the iterative estimation of the response overlaps, the powers of estimates $\widehat{ov}_p^k(t)$ and $\widehat{ov}_s^k(t)$ increased along iterations because of cumulative convolutions with distributions that were not normalized. Consequently this modified ADJAR algorithm did not converge, as shown in Figure 10 illustrating the ratio expressed in dB, between the power of $\hat{a}^{Adjar}(t)$ and $\hat{a}^{Av}(t)$ against the number of iterations. For the original ADJAR algorithm, this ratio converged to a finite value. However when all fixations inside each epoch were considered, this ratio diverged when the number of iterations increased due to iterative convolutions with distributions $(Q_{-}(t), Q_{+}(t))$, which were not normalized to one.

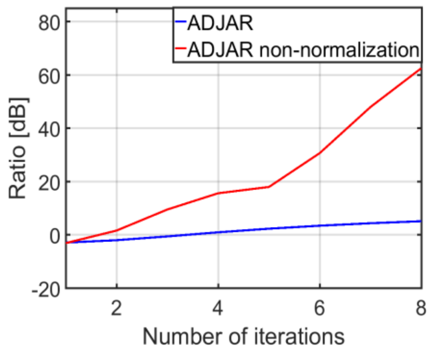

Figure 10 Ratio [dB] between the powers of $\hat{a}^{Adjar}(t)$ and $\hat{a}^{Av}(t)$ against the number of iterations for two cases: blue line for the original ADJAR algorithm and red line for the same iterative algorithm considering all fixations with non-normalized distributions

Theoretical Variance for the GLM estimation

Equation (14) can be developed:

$$\hat{\boldsymbol{a}} = (\boldsymbol{D}^{\dagger}\boldsymbol{D})^{-1}\boldsymbol{D}^{\dagger}(\boldsymbol{D}\boldsymbol{a} + \mathbf{n}) \qquad (34)$$

and the variance $Var[\hat{\boldsymbol{a}}]$ is equal to:

$$Var[\hat{\boldsymbol{a}}] = E\left[(\hat{\boldsymbol{a}} - E[\hat{\boldsymbol{a}}])(\hat{\boldsymbol{a}} - E[\hat{\boldsymbol{a}}])^{\dagger}\right]$$

$$Var[\hat{\boldsymbol{a}}] = (\boldsymbol{D}^{\dagger}\boldsymbol{D})^{-1}\boldsymbol{D}^{\dagger}E\left[\mathbf{n}\mathbf{n}^{\dagger}\right]\left((\boldsymbol{D}^{\dagger}\boldsymbol{D})^{-1}\boldsymbol{D}^{\dagger}\right)^{\dagger}$$

$$Var[\hat{\boldsymbol{a}}] = (\boldsymbol{D}^{\dagger}\boldsymbol{D})^{-1}\boldsymbol{D}^{\dagger}E\left[\mathbf{n}\mathbf{n}^{\dagger}\right]\boldsymbol{D}(\boldsymbol{D}^{\dagger}\boldsymbol{D})^{-1} \qquad (35)$$

with $E[.]$ the mathematical expectation.

The more singular the $\boldsymbol{D}^{\dagger}\boldsymbol{D}$ matrix is, the higher the variance is.

EFRP Estimation in the middle of visual exploration with a larger estimation window

We present here the results obtained in the same condition as in the subsection in Figure 2, for the estimation of EFRP in the middle of visual exploration, but with a larger estimation window [-400; 1550] ms, for the Average method, and the GLM. With such a large window, the ability of the estimation method to separate the precedent and subsequent evoked potentials from the current one was observed. Concerning the estimate given by the Average method, residual activity from the precedent potential was

noted between -400 and -300 ms. This activity was not present for the estimate given by the GLM. Moreover, with this window, the expected return to zero at the end of the estimation window was more visible than with a shorter window. This criterion was only met for the estimate given by the GLM.

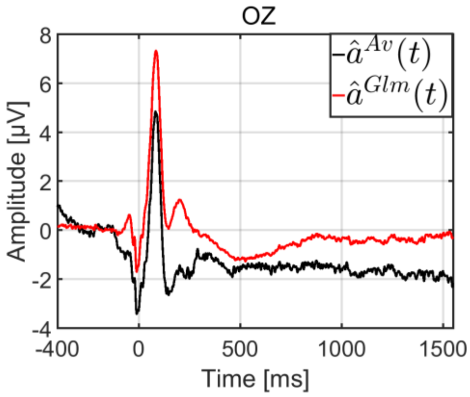

Figure 11 Grand average for the two methods: Average ($\hat{a}^{Av}(t)$), and GLM ($\hat{a}^{Glm}(t)$) on the OZ electrode
